# Outcomes of a Live Messaging, Blended Care Coaching Program Among Adults With Symptoms of Anxiety: Pragmatic Retrospective Cohort Study

**DOI:** 10.2196/44138

**Published:** 2023-02-01

**Authors:** Jocelynn T Owusu, Pam Wang, Robert E Wickham, Sarah F Smith, Jennifer L Lee, Connie Chen, Anita Lungu

**Affiliations:** 1 Lyra Health Burlingame, CA United States; 2 Department of Psychological Sciences Northern Arizona University Flagstaff, AZ United States; 3 Department of Pediatrics Emory University School of Medicine Atlanta, GA United States

**Keywords:** text-based coaching, anxiety, blended care

## Abstract

**Background:**

Anxiety disorders are common and can be debilitating. In addition, various barriers exist that can hinder access to adequate care. Coaching that is grounded in evidence-based interventions and delivered via synchronous (ie, live) text-based messaging could potentially increase the reach of mental health services among populations who select this modality instead of other services (eg, face-to-face coaching and psychotherapy). In addition, the delivery of live messaging coaching within a blended care model has the potential to combine the benefits of coaching with those of evidence-based digital mental health tools.

**Objective:**

This real-world study evaluates the anxiety and satisfaction outcomes of live messaging coaching blended with digital tools (ie, digital exercises and activities).

**Methods:**

This was a retrospective cohort study of 121 adults with moderate levels of anxiety symptoms at the beginning of coaching (Generalized Anxiety Disorder-7 [GAD-7] scores: range 8-14). Participants received an employer-offered blended messaging coaching (BMC) program, and those who opted to receive all live coaching sessions via text-based messaging were included. Anxiety symptom severity was regularly measured by using the GAD-7 scale. Using growth curve models, the change in GAD-7 scores over the course of BMC was evaluated, as were the effects of text-based coaching sessions on GAD-7 scores. The proportion of participants that had a reliable improvement in anxiety symptom severity (GAD-7 score reduction of ≥4) or subclinical symptom severity (GAD-7 score of <8) at the end of care was also estimated. Participants also self-reported their likelihood of recommending their live messaging coach to someone with similar needs.

**Results:**

At baseline, the average GAD-7 score was 9.88 (SD 1.80). Anxiety symptom severity significantly decreased with each week in the BMC program (week: b=−1.04; *P*<.001), and the rate of decline in anxiety symptom severity decreased over time (week^2^: b=0.06; *P*<.001). Each live messaging coaching session was associated with significantly lower anxiety symptom severity during the week of the coaching session (b=−1.56; *P*<.001) and the week immediately following the session (b=−1.03; *P*<.001). Overall, 86% (104/121) of participants had subclinical symptom severity or a reliable reduction in anxiety symptom severity by the end of care. Further, 33.1% (40/121) of participants reported coaching satisfaction levels; of the 40 participants in this subset, 37 (92.5%) were likely or extremely likely to recommend their live messaging coach.

**Conclusions:**

BMC that provides coaching sessions via live messaging can be beneficial for adults with moderate symptoms of anxiety who qualify for and self-select this care modality. Large-scale studies with longer follow-ups are needed.

## Introduction

As some of the most prevalent mental disorders worldwide [1], anxiety disorders are a significant public health problem. Anxiety disorders are associated with various adverse outcomes, including greater workplace presenteeism (ie, working while unwell) and absenteeism [2], as well as the greater use of health care services [3]. Despite the existence of efficacious treatments for anxiety disorders, such as psychotherapy [4], most adults in the United States and around the world do not receive care [5,6]. The prevalence of anxiety disorders, coupled with low rates of care seeking, magnifies the need for innovative treatment approaches that reduce potential barriers to accessing care.

Coaching services have been in use for decades [7] and have become more common in recent years [8]. *Coaching* has been described as a goal-focused approach that fosters development in multiple areas (eg, interpersonal and professional areas) through the collaboration between the coach and the client [9,10]. Although coaching generally does not have a clinical focus [11], coaching programs have been associated with improvements in general well-being (eg, burnout and mental health symptoms) [12], though some results vary [13]. Nonetheless, prior research has also found that live video coaching delivered within a blended care model (ie, video-based coaching combined with internet modules) was associated with significant reductions in anxiety and depression symptoms among adults with moderate symptom severity at the beginning of care [14]. Coaching also holds promise in preventing mental health conditions [15]. There are numerous similarities between coaching and traditional therapy [7], particularly among programs grounded in evidence-based approaches (eg, cognitive behavioral coaching) [16]. Yet, these services differ in several ways. For example, coaching philosophies build on a belief that clients are whole and resourceful and, in practice, place a greater emphasis on person-centered approaches for exploring unrealized strengths in order to maximize self-development [7]. Coaching may also be less stigmatizing than therapy for some [7,9], and stigma is a barrier to mental health care [17]. For these reasons, it is possible that some eligible individuals with mild to moderate levels of psychological distress, including anxiety, may prefer coaching over traditional psychotherapy.

Although coaching can be provided remotely, it is commonly provided in person. Logistical challenges to accessing in-person mental health care can include transportation as well as inconveniences related to timing and location [18]. Telemental health approaches, including text-based care, may address these challenges [19]. Text-based care has the added benefit of potentially allowing for more discretion and greater convenience [20], in addition to possibly allowing for more internal reflection than face-to-face sessions [20,21]. Therapy delivered via synchronous live messaging is associated with improvements in depression and anxiety [20,21]. However, its evidence is limited, and the effectiveness of live text-based coaching is even less established. Additionally, in light of research showing that video-based blended care coaching may improve mental well-being as well as symptoms of anxiety and depression [14,22], there is the possibility that text-based blended care coaching may also be effective for individuals.

Although text-based coaching may provide another care option for those who select this modality instead of currently available treatments for anxiety, there is insufficient evidence on its effectiveness. Therefore, the primary aim of this study was to assess the anxiety outcomes of an evidence-based program, in which coaching sessions were delivered via synchronous messaging (ie, live messaging coaching), among adults with moderate levels of anxiety symptoms. More specifically, this study assessed changes in anxiety symptom severity over the course of a live blended messaging coaching (BMC) program (ie, live messaging coaching combined with digital tools) and at the end of care. As a secondary objective, participants’ satisfaction with their live messaging coach was also evaluated. The results of this study will generate preliminary evidence on the potential utility of a live BMC program as an alternative mode of mental health care delivery for adults living with moderate anxiety symptoms.

## Methods

### Study Overview

This retrospective cohort analysis included adults (aged ≥18 years) who took part in a live BMC program that was delivered by Lyra Clinical Associates with administrative support from Lyra Health. All participants in the BMC program received coaching sessions via live messaging as opposed to video-based coaching sessions. The BMC program was provided to employees and their dependents by employers as a mental health benefit. Participants were employed by 45 unique companies that covered a variety of industry sectors. All participants were self-referred.

### Ethical Considerations

The data used for this retrospective cohort analysis were deidentified, and this study was deemed exempt by the WCG IRB (Western Institutional Review Board-Copernicus Group; Puyallup, Washington). Because data were collected as a part of routine quality control for care offered by Lyra Clinical Associates, participants did not receive any compensation for either their engagement in the coaching program or the completion of assessments.

### Participants

The inclusion and exclusion criteria for the blended care coaching program were described in previous research [14,22]. To summarize, potential participants were routed to the blended care coaching program through a web-based triage process involving the use of predictive modeling, as described elsewhere [22], and completed an initial battery of assessments for establishing baseline severity and appropriateness for services that included the Generalized Anxiety Disorder-7 (GAD-7) scale and the Patient Health Questionnaire-9 (PHQ-9) scale [23,24]. Participants were excluded from the program if their initial GAD-7 score was ≥15, their initial PHQ-9 score was ≥12, or their initial GAD-7 score ranged from 12 to 14 *and* their initial PHQ-9 score was ≥10 [14]. Additional criteria for exclusion included (1) any past hospitalizations for psychiatric reasons, (2) suicidal or homicidal ideation in the past 6 months, (3) current symptoms of posttraumatic stress disorder, (4) extensive alcohol or psychoactive substance use, (5) current violence in a relationship, (6) disclosure of concerns that mandated reporting to an external agency (eg, child or older adult abuse), or (7) individuals currently receiving treatment from a therapist.

**Figure 1:**
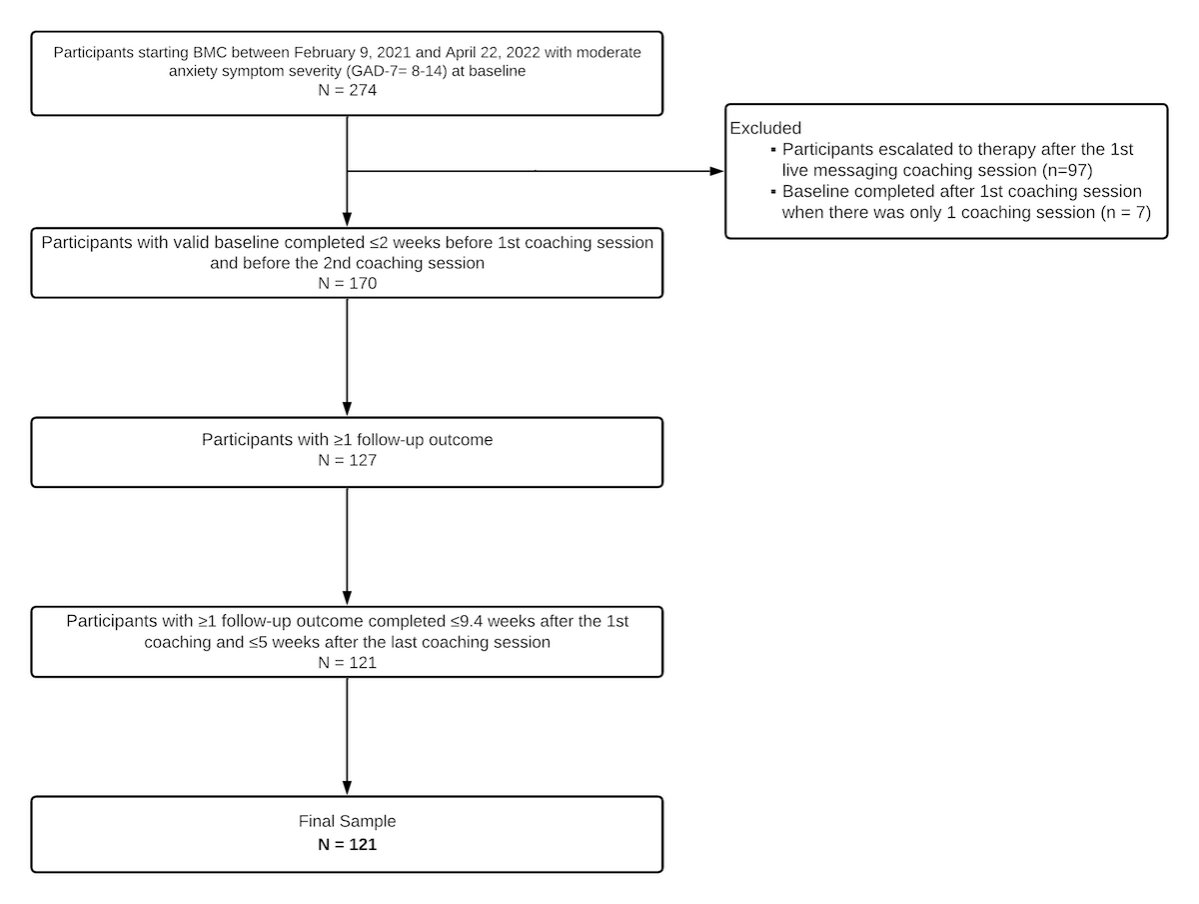
Participant flowchart. BMC participants were defined as those who received all coaching sessions via live messaging over the entire course of the coaching program. BMC: blended messaging coaching; GAD-7: Generalized Anxiety Disorder-7 scale.

Participants self-selected their coaching modality (ie, live messaging or live video-based coaching sessions). This study only included participants who chose to receive all coaching sessions via live messaging (ie, BMC). In addition, only participants with moderate levels of anxiety symptoms were included. Such participants were defined as those who scored ≥8 on the GAD-7 scale [25] and met the additional, previously described GAD-7 criteria on a valid baseline assessment (N=274). Participants were excluded from the analytic sample if they were escalated to therapy after the first live coaching session (eg, care needs were determined to be out of scope for BMC or participant self-referred to therapy; n=97). Of note, baseline GAD-7 scores were not significantly different between participants included in the final analytic sample (mean 9.88, SD 1.80) and those who were escalated to therapy (mean 10.12, SD 1.91; *t*_200.38_=0.95; *P*=.35; Multimedia Appendix 1 provides additional demographics of participants who were escalated to therapy). Participants were also excluded if they did not have a valid baseline GAD-7 score (n=7), did not have any follow-up GAD-7 scores (n=43), or only had invalid follow-up GAD-7 scores (n=6). In order to be included in the analytic sample (ie, valid scores), baseline GAD-7 scores needed to be collected within 2 weeks of the first coaching session and before the second coaching session. Follow-up GAD-7 scores were considered valid only if they were collected ≤5 weeks after the last coaching session. In addition, only ongoing GAD-7 scores that were collected within 1 SD of the average duration of the care received during the BMC program (ie, ≤9.4 weeks after the first coaching session) were included in the analytic sample. The final study sample size included 121 individuals (Figure 1).

### Blended Live Messaging Coaching Program

#### Program Description

**Figure 2:**
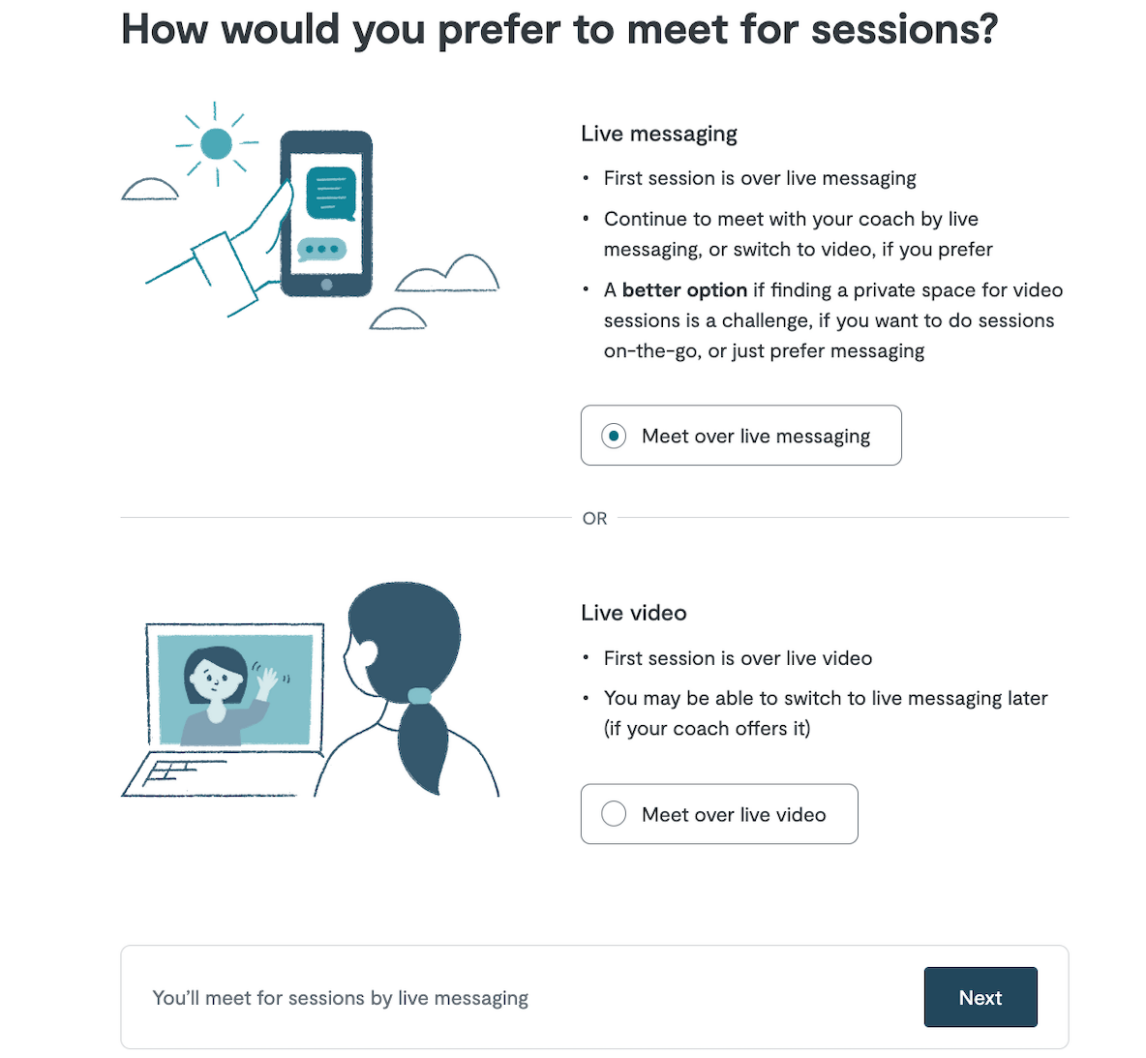
Live messaging coaching platform screen.

The BMC program followed a 6-session model, and participants had the option to extend the program by an additional 6 coaching sessions with clinical approval (eg, because additional coaching goals were identified that were within the scope of the BMC program). Although the intended cadence of sessions was weekly or biweekly, with the tapering of sessions over time, there were no limitations on the duration of the BMC program. However, the overall length of the BMC program was subject to the total number of sessions available as part of the benefits offered by employers, which varied across different companies. Live messaging sessions were scheduled in advance and delivered via synchronous messaging between coaches and participants for a duration of 45 minutes. Participants had the option to transition to video coaching at any time (Figure 2). During live messaging sessions, coaches supported participants who wanted to enhance their mental and emotional well-being and make meaningful changes in their lives through the exchange of text messages. The specific objective of a coaching episode was co-designed by coaches and clients based on individualized needs. Coaches introduced a personalized selection of evidence-based skills and principles derived from cognitive behavioral therapy (CBT) [26], acceptance and commitment therapy (ACT) [27], and mindfulness-based stress reduction [28] while adhering to the core competencies outlined by the International Federation of Coaching, including the use of powerful questioning, planning, and goal setting [29]. The core skills and principles that were potentially offered, which are described in more detail elsewhere [22], included cognitive reappraisal and defusion techniques, values exploration, distress tolerance, mindful awareness, acceptance, opposite action, and effective communication. The introduction of skills and principles was determined by the coach and based on client case conceptualization. For example, when supporting a client struggling with anxiety in social settings, a coach may use cognitive reappraisal techniques to address the catastrophizing of social outcomes; mindful awareness practices to support the observation of internal experiences leading up to, during, and after social interactions; acceptance-based strategies to challenge the avoidance of valued social settings; distress tolerance techniques for in-the-moment coping in high-intensity circumstances; and values exploration as a reason for engaging in anxiety-provoking situations.

Between live messaging sessions, coaches had the option of sharing digital activities, such as video lessons, exercises, and handouts, to enhance in-session learnings and promote the out-of-session practice of key skills. The assignment of digital activities was personalized based on the participant goals and needs identified during the sessions. The activities included a variety of digital video lessons, exercises (eg, digitized versions of CBT worksheets), and handouts that introduced core CBT, dialectical behavior therapy, and ACT principles. Coaches provided written feedback on completed digital exercises and could engage in asynchronous messaging to provide additional support as needed, with an expected response time of 24 to 48 hours.

#### Coaching Training

The blended care coaching training program was described in a prior study [22]. Briefly, all coaches received approximately 60 hours of training in evidence-based techniques during an onboarding phase and participated in ongoing learning and development requirements to maintain mastery of skills. The curriculum comprised live didactic instruction on core intervention skills and case conceptualization, the review of supplemental videos and recorded demonstrations, and skills practice in a small group format and an individual format. Specific attention was given to a case conceptualization approach rooted in the ACT tradition, with a focus on psychological flexibility as a contributor to mental well-being [22]. Coaches were encouraged to think broadly about client concerns, reflect on stuck points within the core intervention skills described above, and identify approaches for skills introduction based on client presentation.

Coaches who offered the live messaging modality received an additional 15 hours of training in the delivery of an effective, impactful coaching session via written text. Building on the foundational curriculum described above, coaches demonstrated competencies in delivering evidence-based techniques in a live messaging format during a dress rehearsal session that was conducted prior to seeing clients. Coaches also received clinical consultations as needed from a clinical team of licensed mental health professionals and participated in ongoing group consultations for the duration of the time in which they offered the modality. The clinical team provided broad oversight of program quality and model fidelity through ad hoc session reviews.

### Measures

Demographic data, including age, gender, and race and ethnicity, were self-reported by participants. Over the course of the BMC program, the GAD-7 scale was delivered to participants prior to scheduled coaching sessions in order to evaluate changes in anxiety symptoms. The GAD-7 scale is a 7-item self-report measure of anxiety symptom severity with strong psychometric properties [23,25]. Each item is scored on a Likert-like scale ranging from 0 (“not at all”) to 3 (“nearly every day”). Total scores on the GAD-7 scale range from 0 to 21, with higher scores indicating greater anxiety symptom severity. A GAD-7 score of ≥8 is an optimal cutoff for identifying those with generalized anxiety disorder [25]. Reliable improvement was defined as a reduction of ≥4 points from the baseline GAD-7 assessment to the final GAD-7 assessment, which aligned with the reliable change index for the GAD-7 scale that was calculated in prior research [30,31]. Recovery was defined as a final GAD-7 score <8, which is indicative of subclinical anxiety symptom severity.

Participants' satisfaction with coaching sessions was evaluated by using the following modified 1-item measure [32]: “How likely are you to recommend your Lyra coach to someone with needs similar to yours?” The response options were “Extremely unlikely,” “Unlikely,” “Neutral,” “Likely,” and “Extremely likely.” This study assessed the responses that were collected after participants’ final live messaging coaching session.

### Statistical Analysis

Baseline descriptive characteristics were calculated. In addition, the average time spent in the BMC program was measured (in weeks), as was the average number of live messaging coaching sessions completed. The average number of sessions completed per week was calculated per person (ie, number of coaching sessions completed ÷ weeks in BMC) and then averaged across the entire sample. Simple change scores (final GAD-7 score − baseline GAD-7 score) were subjected to a 2-tailed paired samples *t* test, and changes in anxiety symptoms across the course of the BMC program were evaluated through a series of individual growth curve models [33], using the lme4 library in R 3.6.3 (R Foundation for Statistical Computing) [34]. Symptom trajectories were modeled by using a linear-quadratic base function (week + week^2^) . In addition, models included participant-level (level 1) random effects for the intercept, linear component (week), and quadratic component (week^2^) of the growth function, as well as a random effect for the intercept at the provider (ie, coach) level (level 2). The association between coaching session engagement and GAD-7 outcomes was also evaluated by entering the number of sessions completed during the previous 7 days and the previous 8 to 14 days as time-varying covariates. Finally, participant age and gender were incorporated as time-invariant covariates for predicting symptoms at baseline.

## Results

### Participant Characteristics

On average, participants were aged 34.00 (SD 9.57; median 33.00; range 19-68) years. A majority of the sample self-identified as female (95/121, 78.5%), while 21.5% (26/121) self-identified as male. Of the 121 participants, 14 (11.6%) self-reported as Asian or Pacific Islander, 4 (3.3%) self-reported as Black or African American, 9 (7.4%) self-reported as Hispanic or Latino, 9 (7.4%) reported multiple racial and ethnic groups, 83 (68.6%) self-reported as White, and 2 (1.7%) did not disclose their race and ethnicity or had an unknown race and ethnicity.

### Engagement and Coaching Satisfaction

Table 1 reports participants’ engagement characteristics and coaching satisfaction levels. The 121 participants who met inclusion criteria attended an average of 3.21 (SD 2.04; median 3.00) coaching sessions and were engaged in care for an average of 3.61 (SD 3.58; median 2.86) weeks. On average, participants completed 0.70 (SD 0.63; median 0.71) coaching sessions per week in BMC. The majority of participants were assigned at least one digital lesson (113/121, 93.4%) or digital exercise (99/121, 81.8%).

Overall, 33.1% (40/121) of participants reported their level of satisfaction with their coach. The mean differences in pre-post GAD-7 scores were not significantly different between those who reported satisfaction levels (mean difference 6.00, SD 3.69) and those who did not (mean difference 4.86, SD 3.66; *t*_77.21_=−1.60; *P*=.11). Among participants who reported satisfaction levels, most were likely (12/40, 30%) or extremely likely (25/40, 62.5%) to recommend their coach to someone with similar needs.

**Table 1:** Blended messaging coaching (BMC) characteristics (N=121).

Characteristics		Value
**BMC duration, mean (SD)**		
	Number of coaching sessions	3.21 (2.04)
	Duration of BMC (weeks)	3.61 (3.58)
	Number of coaching sessions per week in BMC	0.70 (0.63)
Assigned digital lessons, n (%)		113 (93.4)
Assigned digital exercises, n (%)		99 (81.8)
**Likelihood of recommending coaching, n (%)**		40 (33.1)^a^
	Extremely unlikely	1 (2.5)
	Unlikely	0 (0)
	Neutral	2 (5)
	Likely	12 (30)
	Extremely likely	25 (62.5)

^a^Number and proportion of participants that responded to the measure “How likely are you to recommend your Lyra coach to someone with needs similar to yours?”

### Paired Samples *t* Test

Participants reported an average GAD-7 score of 9.88 (SD 1.80) at baseline and an average GAD-7 score of 4.64 (SD 3.41) at their final assessment, resulting in an average change of 5.24 (SD 3.70) points. A paired samples *t* test confirmed that this mean difference was statistically significant (*t*_120_=15.59; *P*<.001), and the standardized effect size was large (Hedges *g*=1.89).

### Growth Curve Modeling

Estimates of fixed effects for the growth curve models are provided in Table 2. Model 1 was composed only of the time variables (week and week^2^) used to specify the growth function, and the corresponding coefficients indicated an estimated GAD-7 score b_intercept_ of 8.47 (95% CI 8.14-8.80) at week 0. The average initial decline of GAD-7 scores was statistically significant (b_week_=−1.43; 95% CI −1.64 to −1.22; *P*<.001), and that decline became significantly shallower (less negative) over time (b_week^2^_=0.11; 95% CI 0.08-0.13; *P*<.001). Models 2 and 3 incorporated the number of coaching sessions attended during the last 7 days and the number of sessions attended during the prior 8 to 14 days as time-varying covariates. Model 2 found that each coaching session attended during the prior week was associated with significantly lower GAD-7 scores (b_sessions 7_=−1.26; 95% CI −1.74 to −0.78; *P*<.001), and model 3 found unique negative associations for sessions attended during the past week (b_sessions 7_=−1.56; 95% CI −2.05 to −1.06; *P*<.001) and sessions attended over the previous 8 to 14 days (b_sessions 8 to 14_=−1.03; 95% CI −1.53 to −0.52; *P*<.001). The inclusion of age and gender in model 3 did not significantly improve model fit, as determined by a likelihood ratio test (*χ*^2^_2_=2.01; *P*=.34).

A likelihood ratio test revealed that including the random effects for the week^2^ coefficient resulted in a significant improvement in model fit, relative to the model 3 specification that included only random effects for the intercept and week terms (*χ*^2^_3_=11.91, *P*=.01). The provider-level (ie, coach-level) random effect was estimated at 0, and likelihood ratio tests indicated that the inclusion of this parameter did not improve model fit (all *P* values ≥.99); however, sensitivity analyses revealed that omitting this effect had no impact on the fixed effect coefficients that were of primary interest. Parameter estimates for random effects are provided in Table 2, and the results of sensitivity analyses for comparing different configurations of random effects are reported in Multimedia Appendix 2.

**Table 2:** Growth curve modeling of anxiety symptoms (Generalized Anxiety Disorder-7 scale).

				Model 1		Model 2		Model 3
**Fixed effects**								
	**Intercept**							
		Coefficient (95% CI)	8.47 (8.14 to 8.80)		8.76 (8.41 to 9.10)		9.00 (8.64 to 9.36)	
		*t*	50.70		50.10		48.97	
		*P* value	<.001		<.001		<.001	
	**Week**							
		Coefficient (95% CI)	−1.43 (−1.64 to −1.22)		−1.31 (−1.53 to −1.10)		−1.04 (−1.29 to −0.80)	
		*t*	−13.09		−11.96		−8.21	
		*P* value	<.001		<.001		<.001	
	**Week^2^**							
		Coefficient (95% CI)	0.11 (0.08 to 0.13)		0.09 (0.06 to 0.12)		0.06 (0.03 to 0.09)	
		*t*	7.20		6.09		3.71	
		*P* value	<.001		<.001		<.001	
	**Sessions (last 7 days)**							
		Coefficient (95% CI)	N/A^a^		−1.26 (−1.74 to −0.78)		−1.56 (−2.05 to −1.06)	
		*t*	N/A		−5.14		−6.19	
		*P* value	N/A		<.001		<.001	
	**Sessions (last 8-14 days)**							
		Coefficient (95% CI)	N/A		N/A		−1.03 (−1.53 to −0.52)	
		*t*	N/A		N/A		−3.97	
		*P* value	N/A		N/A		<.001	
**Random effects, variance (SD)**								
	**Participant-level effects**							
		Intercept	0.909 (0.953)		1.045 (1.022)		1.096 (1.047)	
		Week	0.315 (0.561)		0.339 (0.582)		0.353 (0.594)	
		Week^2^	0.004 (0.063)		0.005 (0.067)		0.005 (0.070)	
	**Provider-level (ie, coach-level) effects**							
		Intercept	0 (0)		0 (0)		0 (0)	
	Residual effects		4.748 (2.179)		4.365 (2.089)		4.177 (2.044)	
Log likelihood				−1150.62		−1138.01		−1130.33
Akaike information criterion				2323.24		2300.02		2286.65
Bayesian information criterion				2369.34		2350.31		2341.13

^a^N/A: not applicable.

### Reliable Improvement and Recovery

Table 3 reports end-of-care reliable improvement and recovery outcomes. By the end of the BMC program, 68.6% (83/121) of participants had a reliable improvement in their symptoms of anxiety (ie, GAD-7 score decreased by ≥4 points from the baseline assessment). Furthermore, 83.5% (101/121) of participants had subclinical levels of anxiety symptoms (ie, GAD-7 score of <8), and 66.1% (80/121) of participants experienced reliable improvement *and* had anxiety symptom severity drop to subclinical levels. Overall, 86% (104/121) had final GAD-7 scores that indicated reliable improvement or recovery.

**Table 3:** Reliable improvement and recovery in symptoms of anxiety (GAD-7^a^ scale).

	Participants (N=121), n (%)
Reliable improvement^b^	83 (68.6)
Recovery^c^	101 (83.5)
Reliable improvement and recovery^d^	80 (66.1)
Reliable improvement or recovery^e^	104 (86)

^a^GAD-7: Generalized Anxiety Disorder-7.

^b^A ≥4 decrease on the final GAD-7 assessment from the baseline GAD-7 assessment.

^c^A final GAD-7 score of <8.

^d^A ≥4 decrease on the final GAD-7 assessment from the baseline GAD-7 assessment and a final GAD-7 score of <8.

^e^A ≥4 decrease on the final GAD-7 assessment from the baseline GAD-7 assessment or a final GAD-7 score of <8.

## Discussion

This study found that a live BMC program was associated with significant improvements in symptoms of anxiety among adults with moderate symptoms at the beginning of care. The effect size of the average pre-post GAD-7 score change was large (Hedges *g*=1.89). In addition, by the end of the coaching program, 86% (104/121) of participants either had a reliable improvement in anxiety symptom severity or experienced a reduction in anxiety symptom severity to subclinical levels. The reliable improvement or recovery rate observed in this study aligned with that of a prior study on the blended care coaching program, which evaluated outcomes when coaching sessions were delivered via live video sessions and found that 88.4% of participants with elevated anxiety symptoms at baseline (ie, GAD-7 score of ≥8) recovered or had a reliable reduction in symptoms by the end of blended care coaching [14]. Our preliminary study suggests that BMC is a comparable option for adults with moderate symptoms of anxiety who opt for this care modality instead of live video blended care coaching.

This study adds to the body of mental health research on synchronous messaging interventions that include text-based therapy [35] by evaluating outcomes of a live messaging coaching program grounded in evidence-based practices. Importantly, the BMC program evaluated in this study complemented live messaging coaching sessions with between-session asynchronous messaging support and digital tools. Because coaching delivered via live messaging may provide an additional treatment option for those with mild to moderate symptom severity, future research should examine the unique components of the coaching modality that contribute to symptom improvement, including differences between synchronous and asynchronous messaging and differences between blended and nonblended live messaging coaching programs.

On average, participants completed 3.2 sessions of coaching over a span of 3.6 weeks. Each live messaging coaching session was associated with statistically significant reductions in anxiety symptoms during the same week of the session and the following week. In addition to the number of sessions, the effects of the quantity and content of the messages exchanged should be examined in future research efforts. These factors could influence coaching outcomes, as well as the relationship between the coach and the client. Prior research has found an association between coaching working alliance and numerous outcomes [36], including participants’ satisfaction with coaching and self-efficacy. In this study, over 90% (37/40, 92.5%) of participants who self-reported coaching satisfaction levels indicated that they were likely or extremely likely to recommend their coach. However, due to the high nonresponse rate, more research is needed to elucidate BMC satisfaction levels. Future studies should also determine the extent to which satisfaction levels influence outcomes of live messaging coaching programs.

The major strengths of this study include the evaluation of live messaging coaching under real-world conditions and the assessment of anxiety symptoms with a validated clinical measure. However, this study had several limitations. Although the observational nature of this study increases the generalizability of the findings, causal statements cannot be made about the live messaging program’s relationship with outcomes in the absence of a randomized controlled design. The sample size was relatively small for a real-world study. Because this program was provided as a mental health benefit to employed individuals and their dependents, the generalizability of the findings to populations outside of this sample are unknown. The generalizability is also limited because a large proportion of the eligible sample was excluded, and the sample was limited to those who remained in BMC for their entire course of care. However, the exclusion of those who were escalated to therapy calls attention to a strength of the program—the ability to triage participants who were not candidates for coaching (eg, those with more severe symptoms) to therapy. Furthermore, a benefit of including those who only received live BMC in this study was the ability to account for potential differential outcomes among those who received more than 1 care modality. As mentioned previously, only 33.1% (40/121) of participants self-reported their level of satisfaction with their coach. Although there was not a statistically significant difference in the average pre-post GAD-7 score change between those who reported coaching satisfaction levels and those who did not (*P*=.11), these findings may still be prone to nonresponse bias. Finally, although ad hoc sessions reviews were performed to assess model fidelity, there were no formal coaching fidelity measures. Therefore, this study was unable to systematically assess the extent to which coaches adhered to evidence-based approaches.

In summary, this study suggests that BMC may be beneficial for adults who are candidates for coaching and present with moderate levels of anxiety symptoms. As a program that provides another care option for those experiencing symptoms of anxiety, a large-scale outcome evaluation is warranted. Future research should also evaluate outcomes after a longer follow-up postcare period and assess program effectiveness for additional conditions (eg, depression and stress). The extent to which working alliance is established and contributes to outcomes in a BMC program also warrants further investigation, especially given the brief duration of BMC and the unique treatment delivery method. Finally, to optimize outcomes, research on program mechanisms of change is also needed.

## Appendix

Multimedia Appendix 1 Participant characteristics of subsample escalated to therapy.

Multimedia Appendix 2 Sensitivity analyses (comparison of growth curve model random effects).

## Abbreviations

ACT: acceptance and commitment therapy
BMC: blended messaging coaching
CBT: cognitive behavioral therapy
GAD-7: Generalized Anxiety Disorder-7
PHQ-9: Patient Health Questionnaire-9

## Data Availability

Because the blended messaging coaching program is an employer-offered mental health benefit, the data sets generated and analyzed during this study are not publicly available.
