# Supplementary material for: Outcomes of a Live Messaging, Blended Care Coaching Program Among Adults With Symptoms of Anxiety: Pragmatic Retrospective Cohort Study
Source: JMIR Form Res. 2023 Feb 1;7:e44138. doi: 10.2196/44138 (PMC9932875; doi:10.2196/44138)
Supplement: Multimedia Appendix 1 [file formative_v7i1e44138_app1.docx]

**Appendix 1.** Participant characteristics of sub-sample escalated to therapy.

|  | **N=97** |
| --- | --- |
| **Age**, Mean (Standard Deviation) | 35.09 (11.53) |
| **Gender**, n (%) |  |
| Female | 73 (75.26) |
| Male | 24 (24.74) |
| **Race & Ethnicity**, n (%) |  |
| Asian or Pacific Islander | 16 (16.49) |
| Black or African American | 8 (8.25) |
| Hispanic or Latino | 9 (9.28) |
| Multiple | 4 (4.12) |
| Other | 1 (1.03) |
| White | 57 (58.76) |
| Prefer Not to Disclose/Missing | 2 (2.06) |
| **Baseline GAD-7**, Mean (Standard Deviation) | 10.12 (1.91) |

**Note**. This sub-sample was excluded from the analytic sample
