# Supplementary material for: Outcomes of a Live Messaging, Blended Care Coaching Program Among Adults With Symptoms of Anxiety: Pragmatic Retrospective Cohort Study
Source: JMIR Form Res. 2023 Feb 1;7:e44138. doi: 10.2196/44138 (PMC9932875; doi:10.2196/44138)
Supplement: Multimedia Appendix 2 [file formative_v7i1e44138_app2.docx]

**Appendix 2.** Sensitivity analyses: Comparison of growth curve model random effects.

|  | **𝛸^2^** | ***P-value*** |
| --- | --- | --- |
| **Participant-level models vs. provider (coach)-level and participant-level models** | *df = 1* | |
| Model 1 | 0.00 | 0.99 |
| Model 2 | 0.00 | 1 |
| Model 3 | 0.00 | 0.99 |
| **Provider (coach)-level and participant-level week vs. provider (coach)-level and participant-level week + week^2^** | *df = 3* | |
| Model 1 | 8.74 | 0.03 |
| Model 2 | 10.56 | 0.01 |
| Model 3 | 11.91 | 0.01 |
